# Complete genome sequence of *Campylobacter* sp. GTC17093 isolated from human skin infection

**DOI:** 10.1128/mra.01338-24

**Published:** 2025-05-19

**Authors:** Jun Yonetamari, Masahiro Hayashi, Yoshinori Muto, Kaori Tanaka

**Affiliations:** 1 United Graduate School of Drug Discovery and Medical Information Sciences, Gifu University 12785, Gifu, Gifu, Japan; 2 Division of Clinical Laboratory, Gifu University Hospital 476117, Gifu, Gifu, Japan; 3 Division of Anaerobe Research, Life Science Research Center, Gifu University 12785, Gifu, Gifu, Japan; 4 Institute for Glyco-core Research iGCORE, Gifu University 12785, Gifu, Gifu, Japan; 5 Gifu University Center for Conservation of Microbial Genetic Resource 12785, Gifu, Gifu, Japan; Wellesley College, Wellesley, Massachusetts, USA

**Keywords:** *Campylobacter*, clinical

## Abstract

*Campylobacter*, a microaerophilic, Gram-negative spirillum, has been isolated from both animal and human specimens; however, most identified species are of animal origin. Here, we report the complete genome sequence of a new *Campylobacter* species, consisting of a 1,660,156 bp circular chromosome, isolated from a human skin infection.

## ANNOUNCEMENT

*Campylobacter* species are Gram-negative, curved, or spiral-shaped bacteria, with a single polar flagellum at one or both ends or no flagellum (1). *Campylobacter jejuni* and *Campylobacter coli* are the most studied species, while the epidemiology and clinical roles of less common species remain poorly understood (2, 3). The strain GTC17093 used in this study was submitted for genetic analysis by a hospital located in Aichi, Japan. It was isolated from an epidermal cyst on the right chest of an adult patient during incision and drainage. This research was performed in accordance with the Declaration of Helsinki.

The sample was cultured anaerobically on Brucella HK agar plates (Kyokuto Pharmaceutical Industrial, Tokyo, Japan) with 5% sheep blood and incubated at 37°C for 48 h. The strain was subcultured three times under the same conditions. Genomic DNA was extracted from the pellet using Quick Taq HS DyeMix (TOYOBO Co., Ltd., Osaka, Japan) for all genome analyses.

The genome was sequenced using Oxford Nanopore Technologies long-read sequencing and Illumina short-read sequencing (4–6). For long-read sequencing, a library was prepared using the SQK-LSK-110 ligation kit (Oxford Nanopore Technologies [ONT]) without shearing, and small fragments were removed using a Short Read Eliminator XS (Circulomics, Baltimore, USA). Sequencing was performed on a GridION X5 (7) with a FLO-MIN106 flow cell (ONT). Guppy v.7.0.9 (super accuracy mode) was used for base calling, and reads were filtered using NanoFilt v.2.7.1 (8) with “-l 1000 -q 10 –headcrop 50.” For short-read sequencing, a library was prepared using an Illumina DNA Prep (M) Tagmentation Kit, and 2 × 151 bp paired-end sequencing was performed on a NovaSeq 6000 platform (Illumina, USA). Reads were processed using fastp v.0.20.1 (6) with “-q 30 -n 20 -t 1 -T 1.” Read quality was checked using Fastp and NanoPlot v.1.32.1 (8). Sequences were assembled using Unicycler v.0.4.8 (9) with default settings. The assembly was rotated to start with the dnaA gene, and Blobtools v.1.0 (10) was used to confirm data integrity. Average nucleotide identity (ANI) analysis was conducted using PyANI v.0.2.12 (ANIm algorithm) (11).

**Fig 1:**
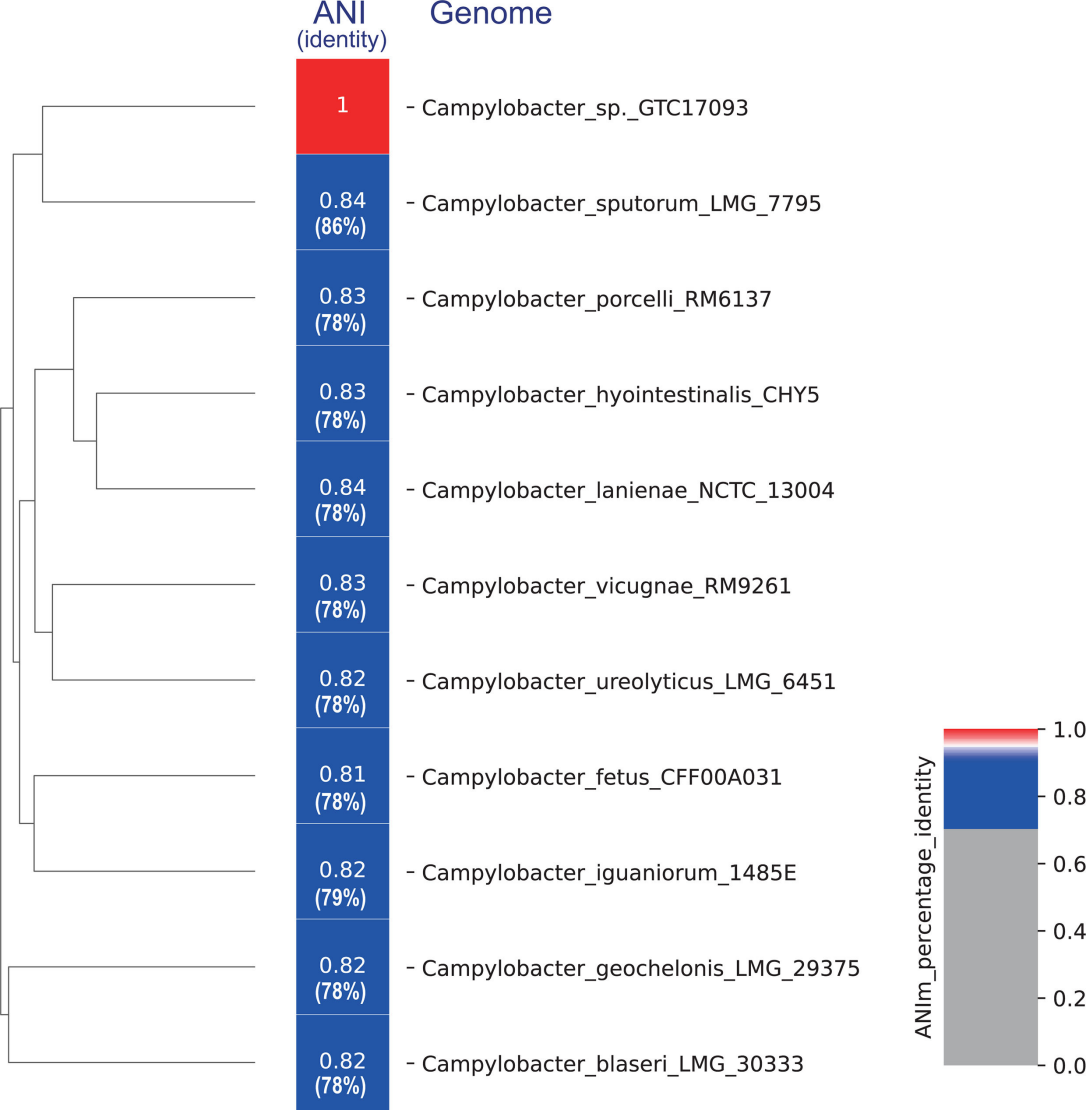
ANI heatmap, generated using PyANI (v.0.2.12) employing the ANIm algorithm, for GTC17093 genome and top 10 closest genomes identified via BLASTN analysis of the whole-genome sequence. The ANIm values are presented in the heat map; sequence identity values obtained via BLASTN analysis are indicated in the brackets.

Genomic data are summarized in Table 1. Genome completeness and contamination were assessed using CheckM (v1.2.2), confirming 100.0% completeness and 0.0% contamination. The DDBJ Fast Annotation and Submission Tool (12) predicted 1,665 coding sequences, nine ribosomal RNAs, 46 transfer RNAs, and one CRISPR. Analysis of the 16S rRNA gene obtained from the genome sequence showed that *Campylobacter sputorum* LMG 7795 was the closest strain, with 98.1% identity. However, species identification with the genome sequence using GTDB-tK (v.2.3.2) was unsuccessful. BLASTN analysis of the complete genome sequence revealed that GTC17093 is most closely related to several *Campylobacter* species with ANI values between 81% and 84% (Fig. 1). The highest ANI value (84.0%) was observed with *Campylobacter sputorum* LMG 7795 (GenBank accession number: GCA008245005.1). However, the strain could not be decisively classified at the species level.

**TABLE 1:** Information regarding the complete genome sequence of *Campylobacter* sp. isolated from a human clinical non-diarrheal specimen

		Strain name
Parameter		*Campylobacter* sp. GTC17093
		GTC17093
Illumina sequencing^a^		
No. of reads		1,921,262
Size (kb)		284,813
Avg coverage (x)		172
DRA accession no.		DRR621827
ONT sequencing^a^		
No. of reads		170,285
Size (kb)		1,243,671
Avg read length (bp)		10,808
Avg coverage (x)		749
N50		13,944
DRA accession no.		DRR621828
Assembly		
Estimated genome completeness (%)^c^		100.0
Estimated genome contamination (%)^c^		0.0
Genome structure		one chromosome
DDBJ/GenBank accession no.		AP038832
Genome size (bp)		1,660,156
GC content (%)		30.4
No. of coding sequences^b^		1,665
Number of rRNAs^b^		9
Number of tRNAs^b^		46
Number of CRISPRs^b^		1

^a^ DRA: DDBJ Sequence Read Archive.

^b^ DFAST and DDBJ fast annotation and submission tools.

^c^ Determined using CheckM.

## Data Availability

The genome sequence of *Campylobacter* spp. (GTC17093) was deposited in the DDBJ (https://www.ddbj.nig.ac.jp/index.html) under the accession number AP038832. Raw sequence data for GTC17093 were deposited in the DDBJ Sequence Read Archive under accession numbers DRR621827 and DRR621828.
